# Whole-Body CT in Haemodynamically Unstable Severely Injured Patients – A Retrospective, Multicentre Study

**DOI:** 10.1371/journal.pone.0068880

**Published:** 2013-07-24

**Authors:** Stefan Huber-Wagner, Peter Biberthaler, Sandra Häberle, Matthias Wierer, Martin Dobritz, Ernst Rummeny, Martijn van Griensven, Karl-Georg Kanz, Rolf Lefering, the TraumaRegister DGU

**Affiliations:** 1 Department of Trauma Surgery, Klinikum rechts der Isar, Technical University Munich, Munich, Germany; 2 IFOM – Institute for Research in Operative Medicine, University Witten/Herdecke, Faculty of Health, Cologne, Germany; 3 Department of Trauma Surgery – Campus Innenstadt, Munich University Hospital, Munich, Germany; 4 Institute of Radiology, Klinikum rechts der Isar, Technical University Munich, Munich, Germany; 5 Committee on Emergency Medicine, Intensive Care and Trauma Management of the German Trauma Society, Berlin, Germany; Università Vita-Salute San Raffaele, Italy

## Abstract

**Background:**

The current common and dogmatic opinion is that whole-body computed tomography (WBCT) should not be performed in major trauma patients in shock. We aimed to assess whether WBCT during trauma-room treatment has any effect on the mortality of severely injured patients in shock.

**Methods:**

In a retrospective multicenter cohort study involving 16719 adult blunt major trauma patients we compared the survival of patients who were in moderate, severe or no shock (systolic blood pressure 90–110,<90 or >110 mmHg) at hospital admission and who received WBCT during resuscitation to those who did not. Using data derived from the 2002–2009 version of TraumaRegister®, we determined the observed and predicted mortality and calculated the standardized mortality ratio (SMR) as well as logistic regressions.

**Findings:**

9233 (55.2%) of the 16719 patients received WBCT. The mean injury severity score was 28.8±12.1. The overall mortality rate was 17.4% (SMR  = 0.85, 95%CI 0.81–0.89) for patients with WBCT and 21.4% (SMR = 0.98, 95%CI 0.94–1.02) for those without WBCT (p<0.001). 4280 (25.6%) patients were in moderate shock and 1821 (10.9%) in severe shock. The mortality rate for patients in moderate shock with WBCT was 18.1% (SMR 0.85, CI95% 0.78–0.93) compared to 22.6% (SMR 1.03, CI95% 0.94–1.12) to those without WBCT (p<0.001, p = 0.002 for the SMRs). The mortality rate for patients in severe shock with WBCT was 42.1% (SMR 0.99, CI95% 0.92–1.06) compared to 54.9% (SMR 1.10, CI95% 1.02–1.16) to those without WBCT (p<0.001, p = 0.049 for the SMRs). Adjusted logistic regression analyses showed that WBCT is an independent predictor for survival that significantly increases the chance of survival in patients in moderate shock (OR = 0.73; 95%CI 0.60–0.90, p = 0.002) as well as in severe shock (OR = 0.67; 95%CI 0.52–0.88, p = 0.004). The number needed to scan related to survival was 35 for all patients, 26 for those in moderate shock and 20 for those in severe shock.

**Conclusions:**

WBCT during trauma resuscitation significantly increased the survival in haemodynamically stable as well as in haemodynamically unstable major trauma patients. Thus, the application of WBCT in haemodynamically unstable severely injured patients seems to be safe, feasible and justified if performed quickly within a well-structured environment and by a well-organized trauma team.

## Introduction

Trauma is one of today's most relevant health issues. In 2009, a total of 177154 deaths in the US were classified as injury-related [1], [2]. With a rate of 184.4 deaths/100000 population, accidents (unintentional injuries) were the leading cause of death up to the age of 54 in 2009 [1], [2]. Beside preclinical therapy and transportation, operative and intensive care unit treatment, early in-hospital trauma management is crucial for the survival of major trauma patients [3]. Therefore, an early, accurate and rational diagnostic workup is necessary. Whole-body computed tomography (WBCT) can be part of such a workup. According to the 2011 annual report of the TraumaRegister of the German Trauma Society (TraumaRegister DGU®) an increasing number of up to 59.5% of the central European trauma centres use WBCT [4]. Its feasibility, speed and accuracy have been proven in several studies during the last decade [5]–[15].

Recently, it could be demonstrated that integration of WBCT into early trauma care significantly increased the probability of survival of patients with polytrauma [16]–[20]. However, the application of WBCT in haemodynamically unstable trauma patients is rejected by many experts. The ATLS® handbook for example states that “CT is a time consuming procedure that should be used only in patients with no haemodynamic abnormalities” [21]. Some warn about CT as a “tunnel-to-death” [22].

The disadvantages for unstable patients are that it could be difficult to escalate care in many CT scanner rooms where access to the patient is poor, lighting is bad, resuscitation equipment is less available, and it may require transporting patients to other parts of the hospital. Clearly these risks depend on local protocols and practice patterns. The advantages of WBCT are earlier diagnosis and targeted, priority orientated treatment planning.

To the best of our knowledge, however, there is so far no evidence that WBCT has any negative or positive effect on the outcome of severely injured patients in shock. Therefore, the above-mentioned reluctance is mainly based on personal opinions and dogmatic tradition. Based on our findings of 2009 in which 85% of the investigated patients were haemodynamically stable, we assessed whether WBCT during trauma-room treatment has any effect on the mortality of shocked trauma patients [17]. Therefore we hypothesized that WBCT has a positive effect on the mortality in haemodynamically unstable trauma patients.

## Methods

### Data collection

We acquired our data from the TraumaRegister DGU® which was started in 1993. It comprises data of major trauma patients of 216 trauma centres mainly from German-speaking countries (Germany, Austria, Switzerland, but also Netherlands, Belgium, and Slovenia; as in 2009)*. It is a prospective, multicentric, standardized and anonymised data base. Every trauma patient admitted to one of the participating trauma hospitals with an injury severity score (ISS) ≥16 or ICU treatment is documented in the registry. Data are continuously entered into a web-based data server that is hosted by the German Trauma Society and its Academy for Trauma Surgery (AUC). Irreversible data anonymity is guaranteed both for the individual patients and the participating hospitals. The registry comprises epidemiologic, physiologic, laboratory, diagnostic, operative, interventional and intensive care medical data as well as scoring and outcome data [23].

The specific parameter “WBCT” has been recorded since 2002. We therefore analysed the database from 2002 to 2009 containing 39.983 patients (TR project ID 09007).

Inclusion criteria were adult blunt trauma patients (age >16 years), ISS ≥16, and available information about the Revised Injury Severity Classification (RISC) score, WBCT during trauma room treatment and the systolic blood pressure on hospital admission. Only those patients were included who were admitted directly from the incident scene and not transferred from other hospitals. Patients who died or received emergency surgery within the first 30 minutes after arrival at the hospital were excluded due to a possible “immortal time bias” [24], [25]. As WBCT is performed about 30 minutes after hospital admission, both groups (shock vs. non-shock) had to have the same chance to reach CT or WBCT to be methodologically comparable in a reliable way.

This study has received the full approval of the ethics committee of the medical faculty of Technical University Munich (TUM), Germany. As the data in the TraumaRegister DGU® are anonymised and routinely collected clinical data obtained from the patients chart no written consent was given by the patients. This has been waived by the approving ethics committee of the medical faculty of Technical University Munich (TUM), Germany (Project number 5340/12). There was no funding for this study.

WBCT is defined as unenhanced CT of the head followed by contrast-enhanced CT of the chest, abdomen, and pelvis, including the complete spine. It can be conducted as single-pass or segmented WBCT. By contrast, no CT or only dedicated CT of one or combined body regions was defined as non-WBCT.

Shock has been defined as follows: Moderate shock as systolic blood pressure of 90–110 mmHg at hospital admission, severe shock as systolic blood pressure of <90 mmHg at hospital admission and no shock as systolic blood pressure of >110 mmHg at hospital admission [26], [27].

The participating hospitals were free to choose their own diagnostic algorithms depending on the type of CT scanners and local emergency department protocols. Neither information about the location of the CT scanner (in or near the trauma room or in the department of radiology) nor information about the specific local CT protocols is recorded in the trauma registry. Due to the high number of 216 participating hospitals in this study, the recorded data can be interpreted as representative for the currently practised standard of trauma care.

### Statistical analysis

Firstly, we performed a descriptive data analysis to compare patients who received WBCT to those who did not using χ^2^ test and Mann-Whitney-U-test (both two sided).

Secondly, we performed a descriptive data analysis to compare patients who were in shock (moderate, severe) with those who were not using χ^2^ test and Mann-Whitney-U-test (both two sided).

Thirdly, we performed an outcome analysis by calculating the RISC score and the standardized mortality ratio (SMR, observed/expected mortality) of those patients who underwent WBCT compared to those who did not undergo WBCT (non-WBCT).

Fourthly, we performed an outcome analysis by calculating the RISC score and the SMR of those patients who were in shock compared to those who were not. SMRs for step three and four were compared using t-test.

The number needed to treat (NNT), or here the number needed to scan, was calculated as follows:

Within each subgroup (moderate, severe, no shock or overall) the difference between RISC prognosis and observed mortality was calculated for both diagnostic groups (WBCT vs. non-WBCT). The absolute risk reduction required for calculating NNT was then derived from these two differences. We chose this way of calculation because the mortality rates in the diagnostic groups were not directly comparable.

Finally, we calculated adjusted logistic regression models in which the effect of WBCT was compared to the well-known prognostic indices of the RISC score in patients with and without shock. The dependent (target) variable was hospital mortality. Further adjustments were made for hospital level (I–III), shock groups (moderate, severe, no shock) and time (years 2002–2009). In an additional logistic regression model, interaction terms (WBCT x shock groups) were included to take account for potential effects of WBCT in the different shock subgroups (moderate, severe or no shock). Furthermore, an additional model was calculated to adjust for potential centre-effects among the 216 participating hospitals.

Details on the methodology and the RISC score have been reported previously [17], [28]. The RISC score is one of the most precise trauma outcome prediction models expressed by its area under the curve (AUC) of the receiver operator characteristic (ROC) of 0.906 (95%CI 0.895–0.918). The components of the RISC score are shown in table 1. Survival was defined as survival to discharge.

**Table 1:** The Revised Injury Severity Classification (RISC) – Score.

Variable	Unit	Value	Coefficient
**Age**	years	55–64	−1.0
		65–74	−2.0
		≥75	−2.3
**New ISS**	points	1–75	−0.03
**Head injury**	AIS points	4	−0.5
		5/6	−1.8
**Limb injury**	AIS points	5	−1.0
**GCS**	points	3–5	−0.9
**PTT**	seconds	40–49	−0.8
		50–79	−1.0
		≥80	−1.2
**Base excess**	mmol/L	−9.0 to –19.9	−0.8
		<–20	−2.7
**Cardio respiratory arrest**	1 = yes 2 = no	1	−2.5
Bleeding signs*	number	1	−0.4
		2	−0.8
		3	−1.6
**Constant**	-	-	5.0

New ISS  =  new injury severity score; GCS  =  Glasgow coma scale; PTT  =  partial thromboplastin time; * Systolic blood pressure <90 mmHg/haemoglobin level <9 g/dL/≥10 units of packed red blood cells; The RISC score is used to calculate the probability of death of a trauma patient. It is one of the most precise prognostic major trauma score [28], [44], [45].

The RISC score, a tool for the calculation of the probability of death of major trauma patients, was used to compute the standardized mortality ratio (SMR), defined as a quotient of the observed to the expected mortality. We calculated 95%CIs when appropriate. Significance was assessed at p≤0.05. The statistical analysis was performed using SPSS (version 20.0).

## Results

### Descriptive Analysis

**Figure 1:**
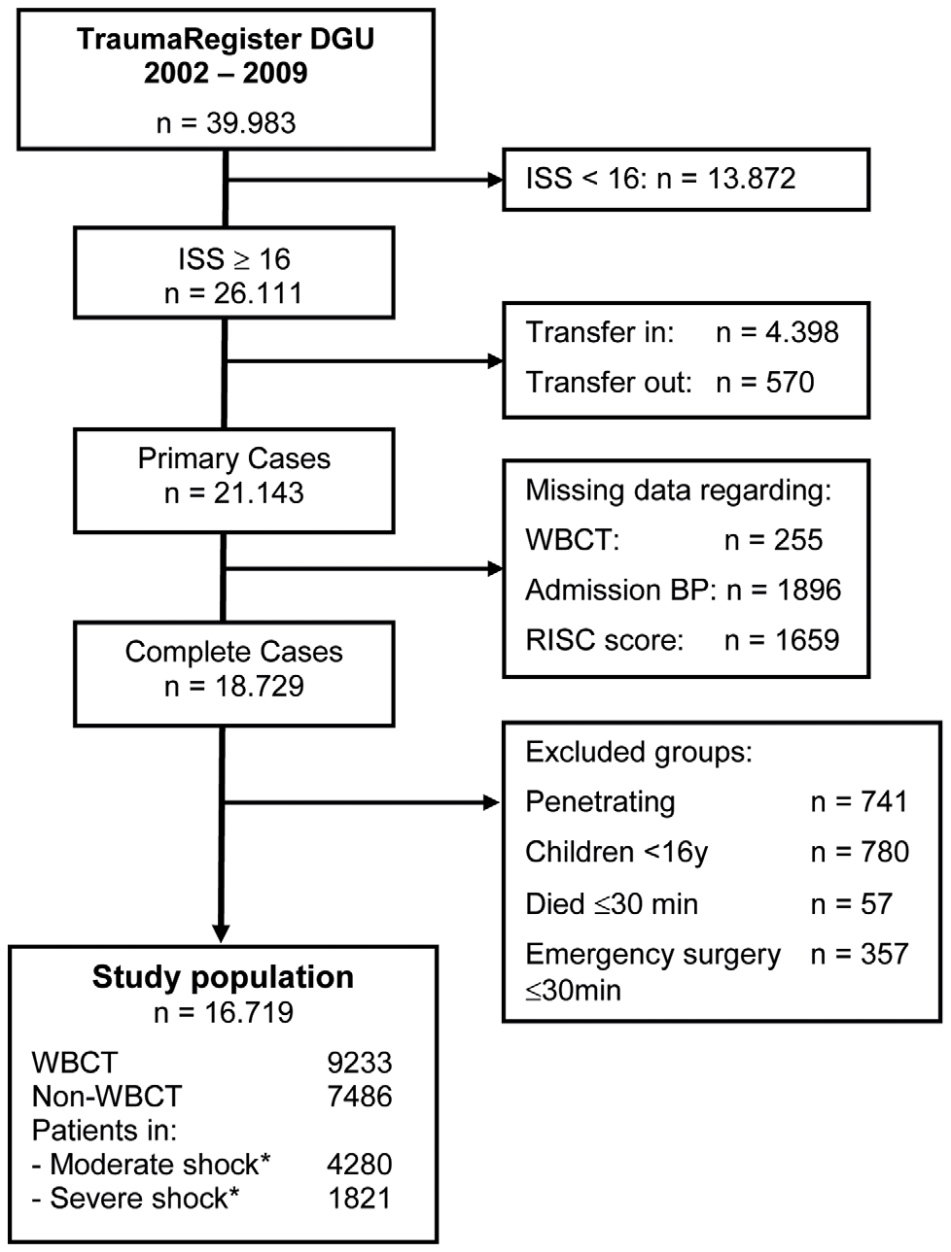
STROBE diagram of study population. ISS  =  Injury severity score, WBCT  =  whole-body CT, BP  =  blood pressure on hospital admission; RISC  =  revised injury severity classification score; transfer in patients: no data of resuscitation in primary hospital; transfer out patients (<48 h): no outcome data of final hospital; missing and excluded groups: overlapping numbers possible; * moderate shock on hospital admission: BP  = 90–110 mmHg; * severe shock on hospital admission: BP <90 mmHg.

16719 of 39983 patients met the inclusion criteria. 9233 (55.2%) of the 16719 patients received WBCT during the early resuscitation phase, 7486 (44.8%) did not (see STROBE diagram, figure 1).

1072 patients (14.3%) assigned to non-WBCT did not undergo any kind of CT investigation until ICU admission and 6414 (85.7%) received selective organ (or combinations thereof) CTs (5653 [88.1%] head, 2689 [41.9%] thorax, 2310 [36.0%] abdomen, 1635 [25.5%] pelvis and 3067 [47.8%] spine).

We did not determine any adverse effects that could be attributed to WBCT. The main characteristics of the investigated patients are given in table 2. The mean time from trauma room admission until CT/WBCT was significantly shorter than for non-WBCT (table 2).

**Table 2:** Characteristics of severely injured patients with information about CT during trauma room treatment.

Group	Total	whole-body CT	Non-whole-body CT	p
**Number**	**16719**	**9233 (55.2%)**	**7486 (44.8%)**	
** Epidemiologic**				
Age (years)	45.8±20.1	45.2±19,8	44.6±20.4	<0.001
Men	12222 (73.1%)	6743 (73.0%)	5479 (73.2%)	0.770
** Prehospital**				
Intubation	9563 (57.2%)	5512 (59.7%)	4051 (54.1%)	<0.001
GCS on scene (points)	10.5±4.8	10.4±4.7	10.5±4.8	0.410
** Trauma Room/in hospital**				
Haemoglobin concentration (mg/dL)	11.7±2.8	11.6±2.8	11.9±2.9	<0.001
Thromboplastin time (%)	77.9±23.4	76.8±23.2	79.3±23.6	<0.001
Base excess (mmol/L)	−3.6±5.0	−3.7±4.8	−3.5±5.1	0.007
Chest x-ray	10989 (65.7%)	4799 (52.0%)	6190 (82.7%)	<0.001
Pelvic x-ray	8588 (51.4%)	3493 (37.8%)	5095 (68.1%)	<0.001
Time from hospital admission to CT (min)	28.5±21.7	24.6±18.0	35.2±25.6	<0.001
Operation rate	12727 (76.1%)	7095 (76,8%)	5632 (75.2%)	0.015
Transfusion of PRBC (any amount)	4798 (28.7%)	2728 (29.6%)	2070 (27.7%)	0.009
Massive blood transfusion until ICU ( ≥10 PRBC transfused)	1187 (7.1%)	697 (7.6%)	490 (6.6%)	0.011
Multiorgan failure*	5484 (32.8%)	3359 (36.4%)	2125 (28.4%)	<0.001
Ventilation time (days)	7.7±11.9	8.1±12.4	7.1±11.1	<0.001
ICU stay (days)	12.0±14.1	12.7±14.7	11.0±13.3	<0.001
Hospital length of stay (days)	26.4±27.9	26.7±27.5	26.0±28.4	<0.001
AIS head ≥3	9915 (59.3%)	5229 (56.6%)	4686 (62.6%)	<0.001
AIS thorax ≥3	9532 (57.0%)	5873 (63.6%)	3659 (48.9%)	<0.001
AIS abdomen ≥3	3413 (20.4%)	2034 (22.0%)	1379 (18.4%)	<0.001
AIS extremities ≥3	6213 (37.2%)	3760 (40.7%)	2453 (32.8%)	<0.001
ISS (points)	28.8±12.1	29.7±12.2	27.7±11.9	<0.001
RISC-Prognosis of death	3528 (21.1%)	1891 (20.5%)	1637 (21.9%)	0.014
24 h mortality rate	1714 (10.3%)	818 (8.9%)	896 (12.0%)	<0.001
Overall mortality rate	3210 (19.2%)	1607 (17.4%)	1603 (21.4%)	<0.001

Data are given as number of patients (% of total patients) or mean ± SD, unless indicated otherwise. SBP Systolic blood pressure, GCS Glasgow Coma Scale; ICU Intensive Care Unit; PRBC packed red blood cells; ISS Injury Severity Score; AIS abbreviated injury scale. RISC  =  revised injury severity classification score; p-value (comparison of WBCT vs. non-WBCT group): χ^2^ test or Mann-Whitney-U test (two sided), *MOF, defined as organ failure of two systems of >2 SOFA-score points of at least 2 days duration [46].

4280 (25.6%) of the 16719 patients were in moderate shock and 1821 (10.9%) in severe shock on hospital admission. 10618 (63.5%) had no shock. The main characteristics of the investigated patients in shock are given in table 3.

**Table 3:** Characteristics of severely injured patients who were in shock compared to those who were not.

	Severe Shock on admission <90 mmHg			Moderate shock on admission 90–110 mmHg			No Shock on admission >110 mmHg		
	1821 (10.9%)			4280 (25.6%)			10618 (63.5%)		
**Whole-body CT (WBCT)**	WBCT 1036 (56.9%)	non-WBCT 785 (43.1%)	p value	WBCT 2462 (57.5%)	non-WBCT 1818 (42.5%)	p value	WBCT 5735 (54.0%)	non-WBCT 4883 (46.0%)	p value
** Epidemiologic**									
Age (years)	46.6±20.2	47.2±20.4	0.54	43.7±19.6	44.6±20.0	0.17	45.6±19.8	47.3±20.6	<0.001
Men	738 (71.2%)	541 (68.9%)	0.29	1711 (69.5%)	1305 (71.8%)	0.10	4290 (74.8%)	3633 (74.4%)	0.62
** Prehospital**									
Intubation	872 (84.2%)	647 (82.4%)	0.31	1719 (69.8%)	1178 (64.8%)	0.001	2925 (51.0%)	2222 (45.5%)	<0.001
GCS on scene (points)	8.1±4.9	7.8±5.0	0.06	10.1±4.8	10.2±4.8	0.38	11.0±4.6	11.1±4.6	0.81
** Trauma Room/in hospital**									
Mean blood pressure (mmHg)	68.1±19.6	61.1±26.1	<0.001	102.0±7.1	103.0±7.2	0.003	139.0±20.2	139.7±20.5	0.001
Haemoglobin concentration (mg/dL)	9.4±3.1	9.0±3.2	0.037	11.1±2.7	11.3±2.8	0.003	12.2±2.5	12.5±2.5	<0.001
Thromboplastin time (%)	59.8±26.2	57.7±27.0	0.12	74.1±23.0	75.4±23.1	0.019	81.0±21.0	83.8±21.2	<0.001
Base excess (mmol/L)	−7.7±6.7	−8.3±7.5	0.14	−4.0±4.4	−4.1±4.5	0.85	−2.7±4.0	−2.5±4.3	0.009
Chest x-ray	548 (52.9%)	613 (78.1%)	<0.001	1295 (52.6%)	1551 (85.3%)	<0.001	2956 (51.5%)	4026 (82.4%)	<0.001
Pelvic x-ray	400 (38.6%)	511 (65.1%)	<0.001	950 (38.6%)	1295 (71.2%)	<0.001	2143 (37.4%)	3289 (67.4%)	<0.001
Time from hospital admission to CT (min)	27.2±20.0	34.1±25.3	<0.001	25.7±18.8	35.3±26.1	<0.001	23.7±17.1	35.3±25.4	<0.001
Operation rate	831 (80.2%)	573 (73.0%)	<0.001	1936 (78.6%)	1455 (80.0%)	0.27	4328 (75.5%)	3604 (73.8%)	0.051
Transfusion of PRBC (any amount)	645 (62.3%)	513 (65.4%)	0.20	918 (37.3%)	665 (36.6%)	0.62	1176 (20.5%)	923 (18.9%)	0.035
Massive blood transfusion until ICU (≥10 PRBC transfused)	278 (26.8%)	198 (25.2%)	0.48	234 (9.5%)	171 (9.4%)	0.88	195 (3.4%)	137 (2.8%)	0.06
Multiorgan failure*	640 (61.8%)	415 (52.9%)	<0.001	1022 (41.5%)	616 (33.9%)	<0.001	1715 (29.9%)	1138 (23.3%)	0.002
Ventilation time (days)	10.4±16.2	7.4±13.5	<0.001	9.7±13.9	8.5±11.9	<0.001	7.1±10.7	6.6±10.4	<0.001
ICU stay (days)	14.4±18.7	10.2±16.0	<0.001	14.6±16.3	12.8±14.3	<0.001	11.6±12.8	10.5±12.4	<0.001
Hospital length of stay (days)	25.7±30.3	21.6±32.8	<0.001	29.3±29.4	30.0±31.7	0.25	25.8±30.0	25.4±26.1	0.002
AIS head ≥3	605 (58.4%)	461 (58,7%)	0.89	1365 (55.4%)	1052 (57.9%)	0.12	3259 (56.8%)	3173 (65.0%)	<0.001
AIS thorax ≥3	785 (75.8%)	510 (65.0%)	<0.001	1624 (66.0%)	958 (52.7%)	<0.001	3464 (60.4%)	2191 (44.9%)	<0.001
AIS abdomen ≥3	378 (36.5%)	255 (32.5%)	0.08	652 (26.5%)	432 (23.8%)	0.043	1004 (17.5%)	692 (14.2%)	<0.001
AIS extremities ≥3	581 (56.1%)	397 (50.6%)	0.02	1154 (46.9%)	708 (38.9%)	<0.001	2025 (35.3%)	1348 (27.6%)	<0.001
ISS (points)	37.9±15.2	37.5±16.5	0.14	31.3±12.5	29.1±12.4	<0.001	27.6±10.6	25.6±9.7	<0.001
RISC prognosis of death	440 (42.5%)	395 (50.3%)	<0.001	524 (21.3%)	400 (22.0%)	0.53	929 (16.2%)	845 (17.3%)	0.010
24 h mortality rate	322 (31.1%)	361 (46.0%)	<0.001	213 (8.7%)	204 (11.2%)	0.005	283 (4.9%)	331 (6.8%)	<0.001
Overall mortality rate	436 (42.1%)	431 (54.9%)	<0.001	446 (18.1%)	410 (22.6%)	<0.001	725 (12.6%)	762 (15.6%)	<0.001

Data are given as number of patients (% of total patients) or mean ± SD, unless indicated otherwise. SBP Systolic blood pressure, GCS Glasgow Coma Scale; ICU Intensive Care Unit; PRBC packed red blood cells; ISS Injury Severity Score; AIS abbreviated injury scale. p-value (comparison of WBCT vs. non-WBCT group): χ^2^ test or Mann-Whitney-U test (two sided), *MOF, defined as organ failure of two systems of >2 SOFA score points of at least 2 days duration [46].

### Outcome Analysis

The recorded mortality rate for **all** 9233 **patients** (shock and non-shock) who underwent WBCT was 1607 (17.4%, 95%CI 16.6–18.2) compared to 1603 (21.4%, 95%CI 20.5–22.3) of all 7486 patients who underwent non-WBCT (p<0.001). A significant difference was also found for the early mortality within 24 hours (see tables 2, 4).

**Table 4:** Standardized mortality ratios – SMRs.

Group	WBCT	Deaths (n)	Overall (n)	Mortality rate (%, CI 95%)	RISC-prognosis	SMR (CI 95%)	p	NNT
**Overall**	yes	1607	9233	17.4 (16.6–18.2)	20.5	0.85 (0.81–0.89)	<0.001	35
	no	1603	7486	21.4 (20.5–22.3)	21.9	0.98 (0.94–1.02)	<0.001	35
**Severe shock BP <90 mmHg**	yes	436	1036	42.1 (39.1–45.1)	42.5	0.99 (0.92–1.06)	0.049	20
	no	431	785	54.9 (51.4–58.4)	50.3	1.10 (1.02–1.16)	0.049	20
**Moderate shock BP = 90–110 mmHg**	yes	446	2462	18.1 (16.6–19.6)	21.3	0.85 (0.78–0.93)	0.002	26
	no	410	1818	22.6 (20.6–24.5)	22.0	1.03 (0.94–1.12)	0.002	26
**No shock BP >110**	yes	725	5735	12.6 (11.8–13.5)	16.2	0.78 (0.73–0.83)	0.003	53
	no	762	4883	15.6 (14.6–16.6)	17.3	0.90 (0.84–0.96)	0.003	53

Data are given as number of patients or % (mortality rate). SMR  =  standardized mortality ratio, BP  =  blood pressure on admission; RISC  =  revised injury severity classification score (mortality prognosis); p-value: t-test (two sided) comparing the two SMRs of each subgroup; NNT  =  number needed to treat or scan.

Expressed as standardized mortality ratio (SMR), the WBCT group reached 0.85 (95%CI 0.81–0.89) compared to 0.98 (95%CI 0.94–1.02) for the non-WBCT group (p<0.001) (see table 4).

The number-needed-to-treat (NNT) or, rather, the number needed to scan related to survival for all patients was 35.

In patients **with moderate shock** on admission, the observed mortality rate for patients with WBCT was 446 (18.1%, 95%CI 16.6–19.6) compared to 410 (22.6%, 95%CI 20.6–24.5) in patients without WBCT (p<0.001). A significant difference was also found for the early mortality within 24 hours (Table 3, 4).

Expressed as standardized mortality ratio (SMR), the WBCT group reached 0.85 (95%CI 0.78–0.93) compared to 1.03 (95%CI 0.94–1.12) for the non-WBCT group (p = 0.002) (see table 4).

The number needed to scan related to survival for patients in moderate shock was 26.

In patients **with severe shock** on admission, the observed mortality rate for patients with WBCT was 436 (42.1%, 95%CI 39.1–45.1) compared to 431 (54.9%, 95%CI 51.4–58.4) in patients without WBCT (p<0.001). A significant difference was also found for the early mortality within 24 hours (Table 3, 4).

Expressed as standardized mortality ratio (SMR), the WBCT group reached 0.99 (95%CI 0.92–1.06) compared to 1.10 (95%CI 1.02–1.16) for the non-WBCT group (p = 0.049) (see table 4).

The number needed to scan related to survival for patients in severe shock was 20.

**Figure 2:**
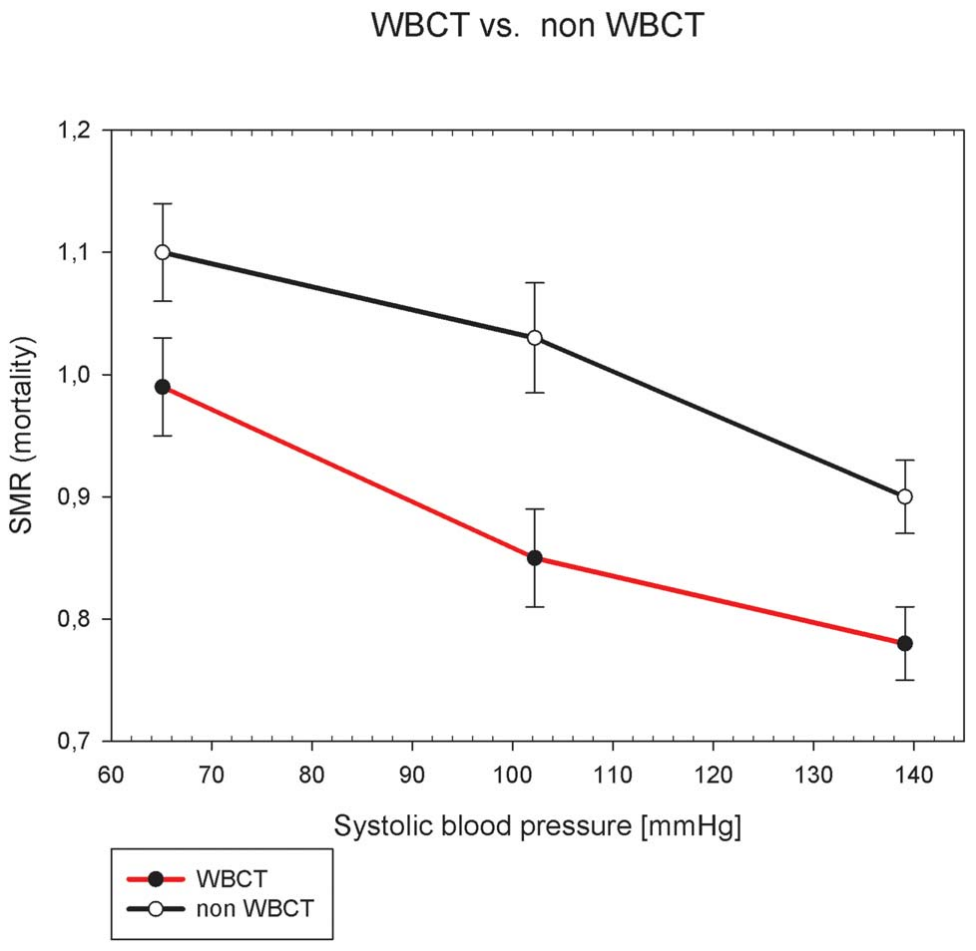
SMR subgroup analysis of the different shock groups at hospital admission (<90, 90-110, and >110 mmHg). The data points are drawn at the mean blood pressure value of the each group. The values of the SMRs are given in table 4. The whiskers show the standard error. SMR  =  standardized mortality ratio.

The results of the SMR analysis are presented graphically in figure 2. All SMRs in all 3 shock categories (moderate, severe or no shock) of the WBCT group are significantly lower compared to the non-WBCT group.

Adjusted logistic regression analysis with the prognostic RISC score showed that WBCT is an independent predictor for survival that significantly increases the chance of survival in all patients (shock and no shock). Adjustment for time (per year) showed that there is a relevant influence on the outcome. Adjustment for the shock groups also showed a relevant effect on the outcome. The effect of adjustment for hospital level (I–III) was not significant. Also the adjustment with interaction terms (WBCT x shock groups) was not significant (see table 5). However, WBCT remained a highly significant and robust independent variable in the model (p = 0.010).

**Table 5:** Adjusted logistic regression model 1, all patients.

Variable	Regression coefficient β	p	Odds ratio (e^β^)	CI 95%
**Model 1: WBCT + RISC, adjusted for hospital level + year + shock subgroup + interactions terms (WBCT x shock groups); all patients (n = 16719)**				
WBCT	−0.18	0.010	0.83	0.72–0.96
Per Year (2002–2009)	−0.65	<0.001	0.94	0.92–0.96
WBCT x no shock (Reference)	-	0.33	-	-
WBCT x moderate shock	−0.15	0.23	0.87	0.68–1.01
WBCT x severe shock	−0.18	0.24	0.84	0.62–1.13
RISC*	0.93	<0.001	2.52	2.44–2.61
No Shock (Reference)	-	<0.001	-	-
Moderate Shock	0.27	0.002	1.31	1.10–1.60
Severe Shock	0.64	<0.001	1.90	1.51–2.38
Hospital level I (Reference)	-	0.064	-	-
Hospital level II	0.13	0.065	1.14	0.99–1.31
Hospital level III	0.34	0.122	1.40	0.91–2.15
Constant	−0.06	0.39	-	-

RISC  =  revised injury severity classification, CI 95%  =  confidence interval; * Inverse logistic transformation of the predicted outcome probability of RISC (mortality).

When logistic regression was calculated within the shock subgroups (moderate, severe and no shock) with adjustment for the RISC score, time (per year) and hospital level (I–III) again WBCT remained stable and highly significant even within each subgroup (moderate shock: OR = 0.73; 95%CI 0.60–0.90, p = 0.002; severe shock: OR = 0.67; 95%CI 0.52–0.88, p = 0.004; see table 6).

**Table 6:** Adjusted logistic regression models 2,3 and 4; shock-subgroups.

Variable	Regression coefficient β	p	Odds ratio (e^β^)	CI 95%
Model 2: WBCT + RISC, adjusted for hospital level + year); no shock patients (n = 10.618)				
WBCT	−0.18	0.014	0.83	0.72–0.96
Per Year (2002–2009)	−0.07	<0.001	0.93	0.90–0.96
Hospital level I (Reference)	-	0.24	-	-
Hospital level II	−0.04	0.71	0.96	0.79–1.17
Hospital level III	0.46	0.11	1.58	0.90–2.75
RISC*	0.98	<0.001	2.67	2.55–2.80
Constant	0.04	0.67	-	-
Model 3: WBCT + RISC, adjusted for hospital level + year); moderate shock patients (n = 4280)				
WBCT	−0.31	0.002	0.73	0.60–0.90
Per Year (2002–2009)	−0.07	0.002	0.93	0.89–0.98
Hospital level I (Reference)	-	0.32	-	-
Hospital level II	0.20	0.14	1.22	0.94–1.58
Hospital level III	0.13	0.76	1.13	0.51–2.54
RISC*	0.87	<0.001	2.38	2.24–2.52
Constant	0.16	0.16	-	-
Model 4: WBCT + RISC, adjusted for hospital level + year); severe shock patients (n = 1821)				
WBCT	−0.40	0.004	0.67	0.52–0.88
Per Year (2002–2009)	−0.034	0.27	0.97	0.91–1.03
Hospital level I (Reference)	-	0.01	-	-
Hospital level II	0.51	0.003	1.67	1.20–2.34
Hospital level III	0.36	0.54	1.43	0.46–4.41
RISC*	0.86	<0.001	2.35	2.18–2.55
Constant	0.37	0.015	-	-

RISC  =  revised injury severity classification, CI 95%  =  confidence interval; * Inverse logistic transformation of the predicted outcome probability of RISC (mortality).

The extent of the effect suggests that the odds of survival in shocked patients could be increased by about 27–33% when WBCT is performed.

Finally, a further logistic regression model was calculated to address potential centre-effects additional to the above-mentioned models. After adjustment for each of the 216 participating hospitals (OR varying from 0.01–19.7), the WBCT effect still remained stable and highly significant (OR 0.72 CI 95% 0.63–8.33, p<0.001; n = 16719, 216 hospitals included).

## Discussion

WBCT during trauma resuscitation significantly increased the survival rate in haemodynamically stable as well as in haemodynamically unstable major trauma patients. Even the probability of survival in patients with shock and WBCT expressed by the SMRs were significantly lower compared to those patients that underwent non-WBCT.

The findings of the recent 2002–2004 analysis could thus be confirmed in the present analysis based on a much higher number of patients [17]. At that time it could be proved that the integration of WBCT into early trauma care significantly increased the *probability* of survival in patients with polytrauma. The absolute mortality rates were statistically not different in this study [17]. In the present study even the absolute mortality rates were significantly lower for the WBCT group compared to the non-WBCT group.

### Comparison with previous studies

The mean ISS of the WBCT (more severely injured) group was two points higher than that of the non-WBCT group. As it could be proven before, this slightly higher ISS based on the diagnoses obtained in the WBCT group is *not* responsible for the increased probability of survival in this group. So, a possible bias, the so-called Will-Rogers Phenomenon is not the explanation for our findings [16], [29].

To the best of our knowledge, there are only a few reports in the literature dealing with the feasibility of WBCT in exsanguinating and thus haemodynamically unstable trauma patients [30], [31]. Additionally, tension pneumothorax, severe brain injury or pericardial tamponade can also cause haemodynamic instability. Many experts reject this kind of diagnostic workup in these patients.

A main argument is the potential loss of time needed to perform WBCT. Optimal time management is crucial for optimal outcome of severely injured patients. As demonstrated by Clarke et al. in 2001, the probability of death increased approximately 1% for each 3 minutes in the emergency department in 243 hypotensive patients bleeding from abdominal injuries needing emergency laparotomies [32]. The time needed to perform a WBCT can currently be estimated to take three to six minutes [7], [10].

We do not state that every unstable major trauma patient must undergo WBCT. There will always be some special circumstances requiring immediate emergency surgery in severely injured patients. We emphasize that the clinical view always needs to be integrated in the decision making process. We also emphasize that in the CT room resuscitation equipment for airway management, ventilation, chest tube insertion, external bleeding control and volume resuscitation should be provided.

To the best of our knowledge, the study at hand provides the first evidence suggesting that WBCT has a positive effect on the outcome even of severely injured patients in shock. In our opinion, WBCT is the best diagnostic tool to detect the cause of shock in an early phase. In 2010 we therefore introduced the term “focused assessment with computed tomography in trauma (FACTT)” [19].

The knowledge of the entire pattern of injuries obtained by early WBCT puts the attending trauma team into the position to address the cause of shock in an optimal, structured and prioritized way. Our data suggest that the decision to operate patients in severe shock is significantly higher in the WBCT group. In the moderate and the non-shock group the operation rates were comparable.

The comprehensive information obtained by whole-body-CT may help to perform more targeted operations and ease the decision for damage control surgery.

The issue whether to apply selective organ CTs or WBCT is still being discussed controversially. Asha et al. found that the associated likelihood of being helped versus harmed was 333.3/12.8 = 26 [33]. In contrast to these findings, Salim and colleagues showed that WBCT resulted in a change of treatment in 19% of 1000 patients without obvious external signs of injuries [12]. Deunk and co-workers reported that additional chest or abdominal CT resulted in a change of treatment in up to 34% of patients with blunt trauma [5].

Smith et al. found that bedside assessment by emergency physicians before CT was sensitive in ruling out serious injuries in high-acuity trauma patients. However, the overall diagnostic accuracy was low, suggesting that “CT should be considered in most high-acuity patients to prevent missing injuries” [34].

We emphasize that the decision to perform WBCT in haemodynamically unstable patients should be made only in hospitals with a well-organized trauma team and appropriate structural requirements, as represented by the hospitals participating at the TraumaRegister DGU®. As we have shown earlier, the concept of WBCT is very well-compatible with the principles of ATLS® [7].

In our opinion, when planning or rebuilding emergency departments, CT scanners should be placed close to or probably best in the trauma room. This is because of logistic reasons. Further research is needed in this field.

According to Stengel this study contributes evidence to the efficiency level of WBCT. In the PATRES-study Stengel et al. investigated the accuracy or efficacy level of WBCT during trauma resuscitation. They found that “single-pass WBCT is highly specific but has a variable sensitivity. Screening tests in trauma are intended to immediately detect life-threatening injuries. Given this premise, high specificity makes WBCT a valuable tool for priority orientated treatment planning.” They conclude that their results on the accuracy/efficacy of WBCT “may help to understand the survival benefit” found in previous studies [35].

### Radiation

The issue of radiation is crucial when discussing the advantages and disadvantages of WBCT. The number of CT examinations increases every year [36] so that CT imaging is made responsible for the increase of radiation exposure which potentially increases the risk of developing cancer [36], [37].

New software algorithms seem to have a great potential for dose reduction. Iterative reconstruction is a better and more accurate way to produce a CT image from the raw data compared to the normal filtered back projection. However, it takes slightly more time to calculate images with iterative techniques. As computer technology improves every year, it is nowadays possible to use these new reconstruction methods within a normal time setting. With these iterative reconstruction techniques it is possible to reduce artefacts and noise in CT images. A reduction of 30 to 80% with iterative reconstruction techniques keeps the same image quality compared to a normal dose setting and filtered back projection images [38]–[40]. Thus, the effective dose of WBCT should no longer be estimated to be around the well-known 10–20 mSv [41], but rather 5–10 mSv, as iterative techniques are becoming more widespread [38]–[40]. Thus, the risk of radiation-related long-term complications is reduced and is outweighed by the positive effects as presented in our study.

Loewenhardt et al. currently demonstrated that the effective dose was 16–22% lower in a series of 100 polytrauma patients when the arms were raised. With the latest design of a 64-slice multi detector CT the effective dose could be reduced even to 10.8 mSv compared 14.3 mSv with a 14-slice multi detector CT (both arms raised; p<0.001) [42].

In our opinion, haemodynamically stable trauma patients should be scanned with arms up and haemodynamically unstable patients with arms positioned alongside the abdomen in order to save time [43].

### Limitations

Additional to the unchanged limitations of our methodology published in 2009 [17], we emphasize that our results show associations rather than causalities. Furthermore, we can just define shock based on the blood pressure documented on admission due to the structure of the TraumaRegister DGU®. The initial blood pressure on admission, however, “stigmatizes” the patient whether he is judged as haemodynamically unstable or stable.

A relevant potential selection bias can be excluded, as the basic characteristics of the groups (age, gender, GCS, and ISS; see table 2 and 3) are almost not significantly different.

Further studies will be necessary to determine exact and valid indications for WBCT in trauma patients. Which patients will profit most from this kind of diagnostic workup? Where is the borderline towards less injured patients? Based on our data we can state that at least “real” severely injured or polytrauma patients probably profit from WBCT as represented by our high mean ISS of 29.

## Conclusions

Based on the analysis of 16.719 patients those with shock on admission and whole-body CT had significantly better survival rates and SMRs compared to those who did not receive whole-body CT. Moreover, we were able to show that the advantage of WBCT during early resuscitation was similar for those with moderate and severe shock compared to those without shock. This may change clinical practice.

Thus, applying WBCT in haemodynamically unstable patients seems to be safe, feasible and justified if conducted quickly within a well-structured environment and by a well-organized trauma team.
